# A Systematic Review of the Guidelines and Delphi Study for the Multifactorial Fall Risk Assessment of Community-Dwelling Elderly

**DOI:** 10.3390/ijerph17176097

**Published:** 2020-08-21

**Authors:** Jieun Kim, Worlsook Lee, Seon Heui Lee

**Affiliations:** 1 Red Cross College of Nursing, Chung-Ang University, Seoul 06974, Korea; jieunk0329@gmail.com; 2 National Evidence-based Healthcare Collaborating Agency, Seoul 04554, Korea; moonlee@neca.re.kr; 3 Department of Nursing Science, College of Nursing, Gachon University, Incheon 13120, Korea

**Keywords:** accidental falls, risk assessment, aged, community health nursing, systematic review, Delphi technique

## Abstract

As falls are among the most common causes of injury for the elderly, the prevention and early intervention are necessary. Fall assessment tools that include a variety of factors are recommended for preventing falls, but there is a lack of such tools. This study developed a multifactorial fall risk assessment tool based on current guidelines and validated it from the perspective of professionals. We followed the Meta-Analysis of Observational Studies in Epidemiology’s guidelines in this systematic review. We used eight international and five Korean databases to search for appropriate guidelines. Based on the review results, we conducted the Delphi survey in three rounds; one open round and two scoring rounds. About nine experts in five professional areas participated in the Delphi study. We included nine guidelines. After conducting the Delphi study, the final version of the “Multifactorial Fall Risk Assessment tool for Community-Dwelling Older People” (MFA-C) has 36 items in six factors; general characteristics, behavior factors, disease history, medication history, physical function, and environmental factors. The validity of the MFA-C tool was largely supported by various academic fields. It is expected to be beneficial to the elderly in the community when it comes to tailored interventions to prevent falls.

## 1. Introduction

Approximately one-third of all people over 65 years of age experience at least one fall, and 15% fall at least twice in their lifetime. [1]. Falls are among the most common causes of injury to the elderly, and they can lead to physical disability, including fractures that result in long-term disability, and reduced exercise capacity; they can even be fatal [2]. The mortality rate for fall-related injuries was 61.6 per 100,000 United States residents aged ≥ 65 years in 2016 [3]. Falls associated with the elderly are also related to the financial burden, not only for the suffering patients but also the increased costs for elderly medical expenses in the health care system. In 2015, costs for falls to Medicare alone totaled over US$ 31 billion in the United States [4]. As falls affect physical, mental, and economic conditions, prevention and early intervention are necessary.

Although there is an increase worldwide in the falls associated with the elderly in the community, the integrated multi-factor assessment tools based on evidence are limited. The limitations of previous fall assessment tools involve the independent identification of physical, psychological, or environmental factors. There were several “physical function” instruments used in the assessment of the risk of falling, which were the Berg Balance Scale, the Timed Up and Go Test, and the Tinetti Balance Assessment [5,6]. However, the Fall Efficacy Scale and the Activity Specific Balance Confidence Scale are tools for assessing “psychological factors” and have attracted attention in assessing the elderly in the community [7]. Regarding “environmental assessment” tools like the “home falls and accident screening tool” and “Westmead home safety assessment,” a number of instruments are available for home safety assessments [8,9]. All of the tools, as mentioned above, have a commonality in predicting the risk of falls using only one or two factors. Several meta-analyses and systematic reviews of fall prevention and tailored intervention programs recommend a fall assessment tool that includes a variety of factors [10,11].

Therefore, this study applied the multifactorial risk model, which is commonly used to predict the risk of aging-related diseases in the community elderly [12,13]. Such multiple factors may increase the real risk of future illness. For proper prevention, it is necessary to consider the full spectrum of individual and environmental levels. This is directly related to reducing the incidence of fall risk in the elderly. High-quality systematic reviews have reported that fall intervention based on multifactorial assessment had the effect of lowering falls (six studies, risk ratio (RR) = 0.67, 95% confidence interval (CI) = 0.55–0.82), whereas single intervention with single-factor assessment did not [14]. The purpose of assessing fall risks in consideration of multiple factors is to provide interventions that take these factors into account. However, the fall-risk assessment tool (FRAT-up), as an existing multifactorial fall risk assessment tool, incorporates information from multiple domains into a single fall risk score [15]. While this is derived by summing the scores of all factors to determine an overall risk of falls, our tool focuses on assessing all items affecting fall risk. This is important because it can provide tailored interventions based on the results of fall risk assessment.

Additionally, various notable organizations have developed guidelines containing recommendations for fall risk screening to provide tailored interventions [16,17,18]. When developing earlier practical guidelines, they were analyzed by synthesizing articles, not guidelines for the fall risk assessment. Guidelines advocate decisions about appropriate health care practices for specific clinical circumstances for practitioners and patients [19]. It is meaningful to review these guidelines as they were developed by comprehensively analyzing the effects of previous studies. However, to date, internationally agreed guidelines for fall risk assessment do not exist. This study revisits the fall risk assessment guidelines based on currently available evidence.

In primary care settings, it is essential to provide a basis for identifying fall risk factors for the assessment. The purpose of this study was to systematically review current multifactorial fall risk assessment guidelines on community-dwelling elderly. Ultimately, this study comprehensively presented all the relevant recommendations for fall risk assessment.

## 2. Materials and Methods

### 2.1. Systematic Review

This study followed the guidelines in the Meta-Analysis of Observational Studies in Epidemiology (MOOSE) statement [20]. Two researchers (KJE and LWS) independently extracted data and evaluated the quality of studies. Disagreements between the researchers were resolved by conducting a joint review with a third researcher (LSH) to reach a consensus. The Institutional Review Board of K University Hospital (IRB NO. ED15350) approved this study.

In this research, the search was concluded on August 18, 2016; however, an update was performed to confirm recent evidence. The final date of the search for all databases was July 25, 2020, with no date limits. We searched the following electronic databases: OVID-MEDLINE, EMBASE, Cochrane Library, Trip database, Guideline International Network, National Guide Clearing House, the World Health Organization (WHO), and Centers for Disease Control and Prevention (CDC). We also searched five Korean databases: Research Information Sharing Service (RISS), Korean Studies Information Service System (KISS), National Assembly Library, Korea Med, and the Korean Medical Database (KM base). Later, we rescreened by searching for the bibliographies of all the related papers. Participants were elderly residing in the community. The type of outcome was factors and/or items of multifactorial fall risk assessment, and the type of study involved guidelines. The search terms are reported in Table S1.

First, two researchers (LWS and KJE) independently reviewed the titles and abstracts of the searched articles. Second, we reviewed the full manuscripts of eligible studies and recorded the reasons for exclusion for each study. The inclusion criteria were as follows: (a) studies in which research subjects were community-dwelling elderly defined as aged 65 and over, (b) studies in which research interventions had a multifactorial fall risk assessment, and (c) studies in which the evidence was based on guidelines only. Exclusion criteria were as follows: (a) studies in which research subjects were in facilities (e.g., hospitals or nursing homes), (b) studies in which research subjects had a specific disease (e.g., community-dwelling elderly with Parkinson’s disease), (c) studies in which the guidelines had interventions but no assessment components, (d) studies not published in English or Korean; (e) studies that did not contain guidelines, and (f) studies for abstract or conference proceedings only.

### 2.2. Delphi Study

We conducted a Delphi study to facilitate consensus among Korean experts. Prior studies on the Delphi research method state that about 10 panelists were needed to minimize errors and maximize reliability or judged that 8–12 people were appropriate [21]. If the number of experts is too small, it is difficult to agree on an adequate number of topics, and if they are too many, it is a time-consuming process. We recruited eleven experts for the Delphi panel. However, nine experts agreed to participate, and two experts refused. All experts who participated in the study were informed about the aims of the study and provided informed consent.

To prepare for the first round, the research team developed indicators for each element of the multifactorial fall risk assessment tool among community-dwelling elderly that originated from the reviewed guidelines. When planning a Delphi study, we set the criteria for the end of the rounds as a completed round for the expert’s consensus, and not as the number of specific rounds [21]. The first round was open. The first Delphi meeting with a multidisciplinary expert panel was held from October 13 to 26, 2016, by e-mail. Experts reviewed opinions about the appropriateness of classification; the necessity to add, correct, delete, and integrate the determinants identified in the systematic review; and the need to change their order. The validity of the Delphi technique was increased using qualified experts [22]. The expert group consisted of a total of nine Ph.D. experts, three geriatric medicine professors, two medical doctors, two nursing professors, one nurse, one police science professor, and all of them had previous fall-related research or practical experience for over five years.

We included scoring beginning with the second round. The second Delphi meeting with the same expert panel was held from 22 December, 2016, to 19 January, 2017, by e-mail. The mean, standard deviation, median, and interquartile range of experts’ opinions about the necessity and applicability dimensions were presented in the questionnaires that followed each round. An expectation of the Delphi process was for the expert group to reach a consensus; this study reached a consensus among experts in the third round. During the three rounds of the Delphi questionnaires, data were collected by e-mail. The experts reviewed opinions and decided the appropriateness of the items. They considered reasons to add, correct, delete, and integrate the items from determinants, as well as changes to the order. In addition, the expert panel was asked to evaluate each item on a 5-point Likert scale (strongly disagree to strongly agree) along the two dimensions of necessity and applicability to the community-dwelling elderly. Data from each round were analyzed, and experts received feedback that presented information, including the written opinions and anonymous results of the ratings.

To select the components of the final questionnaires for the tool, we analyzed additional opinions from the panel of experts. The criteria chosen for scoring the survey were as follows: content validity ratio (CVR) ≥ 0.78 (minimum value for nine panelists), degree of consensus (DoCs) ≥0.75, and degree of convergence (DoCv) ≤0.50. Cronbach’s alpha test was used to determine internal consistency when the criteria were scored higher than 0.7. Furthermore, to evaluate stability, only items with coefficients of variation (CV) of 0.80 or more were deleted [22]. Self-assessment of the research design was conducted to ensure the quality, all of which met its standards. The questions were, “What criteria will be used to determine which items to drop?” and “What criteria will be used to determine to stop the Delphi process?” [21]

## 3. Results

### 3.1. Systematic Review and an Initial List of Potential Standards

**Figure 1:**
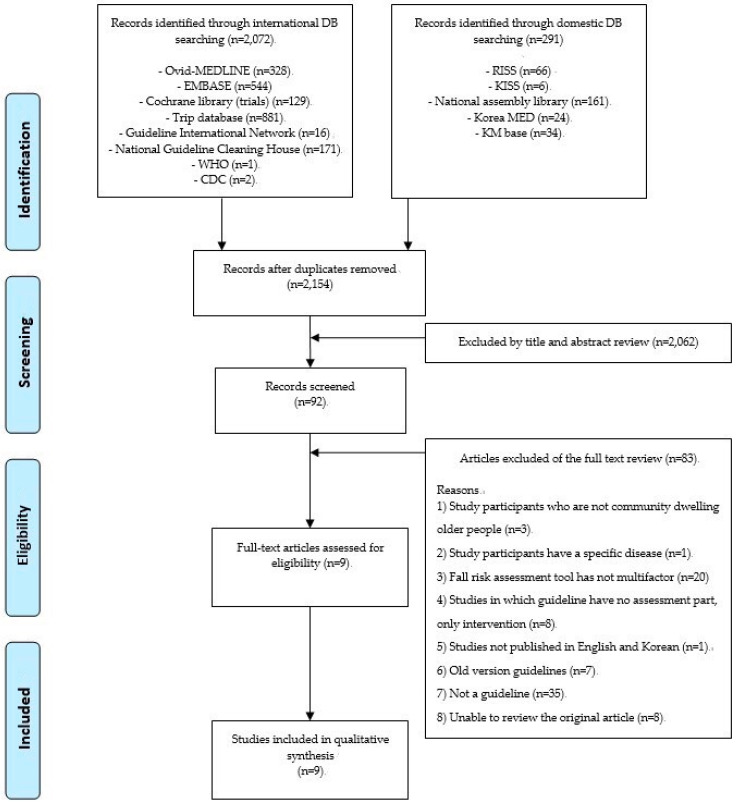
Updated flow chart. Notes: DB = Data Base; WHO = World Health Organization; CDC = Centers for Disease Control and Prevention; RISS = Research Information Sharing Service; KISS = Korean studies Information Service System; KM base = Korean Medical Database.

Figure 1 shows an updated flow chart of the search results, and the previous chart is reported in Figure S1. After updating the search for guidelines, one guideline was added [23]. Of the 2072 articles retrieved by our database search, 92 were selected based on the titles and abstracts. We included a total of nine articles describing guidelines for multifactorial fall risk assessment among community-dwelling elderly [24,25,26,27,28,29,30,31]. The included guidelines are described in Table 1. The nine guidelines are classified by country: two were from Canada [29,30], one from Australia [24], one from Ireland [27], one from the United States of America [23], and the other four guidelines were not restricted by country [25,26,28,31]. Likewise, the participants’ ages in nine of the guidelines were over 65 years. There were no gender restrictions in any of the guidelines. All nine articles were classified by the person who performed the assessment tool: one by the health care provider [28], one by the physical therapist [25], two by health professionals [24,26], one by community health workers [30], one by the primary health care teams [31], one by clinicians [23], and two were not identified [27,29]. The number of factors for each guideline was two to four.

The results of the quality assessment of guidelines, using the Appraisal of Guidelines for Research and Evaluation II (AGREE II), indicated that they ranged from 66.7 to 100.0% (Table 2). The Australian Commission on Safety and Quality in Health Care guidelines scored highest on the overall assessment (100.0%), while all the other guidelines scored 66.7%. The six domain scores of the AGREE II were evaluated separately. The highest scored domain was the “Scope and Purpose” (83.0%), and the lowest scored domain was “Applicability” (36.5%). We discussed the results of the quality assessment and concluded that no guidelines would be excluded when conducting the Delphi study.

The initial factors and items that resulted from our systematic review and the discussion by the researchers are listed in Table 3. We excluded ethnicity (Race), thyroid dysfunction, hearing, risk-taking behavior, and weather and climate from the list of items through the systematic review, because they did not fit due to ambiguity. Altogether, eight items were selected for behavioral factors, 17 for biological factors, three for environmental factors, and two for general factors. Since the factors and items for fall risk in updated guidelines have not been newly added, the Delphi has not been implemented again.

### 3.2. Delphi Study to Identify and Prioritize Standards

#### 3.2.1. Open Round

For the four factors and 30 items chosen, we performed the open round with a panel of experts (nine experts from five fields), providing their thoughts on the suitability of the Multifactorial Fall Risk Assessment Tool for Community-Dwelling Older People (MFA-C) in narrative form. The typical answers related to factors and items needed to be modified, added, reordered, integrated, or moved to other factors. As a result, four factors (behavior, biological, environmental, and general) were reclassified into seven factors (general characteristics, behavior factors, disease history, medication history, physical function, cognitive function, and environment factors), and the existing 30 items were reorganized according to these new factors. At this time, the disease history item was moved to the factor level, and 10 items were added and included in that factor (Table 4).

#### 3.2.2. Consensus in Scoring Rounds

Nine experts from five fields participated in the scoring round. Through the first round (the open round), 39 items under six factors were suggested. The scoring round was conducted twice, and a total of three rounds (one open round and two scoring rounds) were completed in nine months.

In the second round (the first scoring round), expert panelists agreed on 33 out of 39 items (84.6%) (Table 4). The scoring round comprised segments for the necessity and applicability of the scale to community-dwelling elderly. In the necessity segment, the expert panel agreed on CVR, DoCs, DoCv, and CV. In the applicability segment, the CVR value of the medication side effect in the medication history factor was less than 0.79. The low-income item of the general characteristics factor, vitamin D deficiency of the behavior factor, incontinence of the disease history factor, the medication side effect of the medication history factor, the cardiac function of the physical function factor, and the cognitive capacity of the cognitive function were all less than 0.75 for DoCs or higher than 0.50 for DoCv.

Of these six items that corresponded with the exclusion criteria, three items (low income, incontinence, and cardiac function) were re-included based on the expert panel’s judgment. Additionally, all of the CVs were less than 0.80. However, another three items (mediation side effect, vitamin D deficiency, and cognitive capacity) were excluded from this round after reaching an expert consensus. The experts concluded that medication side effects and cognitive capacity were duplicated with the newly added items of the disease history factor. In addition to identifying vitamin D deficiency, a blood test had to be performed. However, the expert panel determined that it would be inappropriate for community workers to assess the risk of falls and that this would place an economic burden on the elderly. In the third round (the second scoring round), the panels reached 100.0% agreement (36 of 36), thereby concluding the scoring round. Therefore, the final version of MFA-C had 36 items in six factors (Table 5).

## 4. Discussion

We systematically reviewed previously distributed individual fall risk factors, thereby facilitating the potential prevention of and early intervention in falls through the development of a multifactorial assessment tool that can be applied practically in the community. To our knowledge, this is the first study to develop a fall risk assessment tool through the Delphi study in various fields based on systematic review results that include multiple fall risk factors in the guidelines published. Previous studies have shown that there are differences in the items for developing a fall risk assessment tool based on the varied experiences of nurses or physicians [32]. Representatively, the tool by the National Health Service (NHS) in Bristol comprises 13 items: history of falls, medications, postural hypotension, alcohol intake, nutrition and osteoporosis, vision, hearing, walking/gait, transfers, function, continence, environmental hazards, and cognition [18]. Compared with the tool provided by the NHS, our tool was developed with more comprehensive and detailed assessment items related to the risk of falling. For a more accurate and in-depth verification of effectiveness using our fall risk assessment tool, systematic reviews of guidelines and confirmation of various expert opinions were necessary.

### 4.1. Items Excluded from this Multifactorial Assessment Instrument

Among the final items presented in this study, we excluded a lack of vitamin D, medication side effects, and cognitive capacity, all of which were considered fall risk items in the existing eight guidelines. Several studies reported that vitamin D reduced the risk of falls, and one meta-analysis estimated a 20% reduction in fall risk through vitamin D supplementation in the elderly [33]. These studies posited that the correlation between low serum 25-hydroxyvitamin D (25(OH)D) and increased falls was due to the lack of 25(OH)D, which leads to muscle weakness and poor balance [34]. As a result, this could lead to decreased physical performance and aging [34]. However, it also indicates that vitamin D deficiency does not have a direct effect on falls, but somewhat weakens the musculoskeletal system, resulting in falls. In this study, the final fall risk assessment tool includes the musculoskeletal function item of the physical function factor. Therefore, the Delphi panelists excluded vitamin D from the risk assessment tool because it was a duplication. In addition, recent studies have shown that supplemental vitamin D did not prevent falls [35], nor did it have a significant correlation with falls [36]. Furthermore, the National Institute for Health and Care Excellence (2013) does not recommend the use of vitamin D for fall prevention because there is a lack of robust evidence regarding the required dosage or method of administration [16]. For this reason, the expert panelists determined that invasive and costly vitamin D testing to assess fall risk was inappropriate for the elderly.

Furthermore, two items (medication side effect and cognitive capacity) in the Delphi phase were excluded because they were considered to overlap with other items of the disease history factor. In particular, the medication side effect item in the existing guidelines did not list specific disease names; therefore, the use of the item to perform a fall risk assessment could reduce the reliability of the evaluation because the results would vary according to the person performing the evaluation.

In this study, only the “fear of falling” was identified as an item related to psychological characteristics. Recent studies have reported that fall-related psychological concerns directly affected falling and its complications [7]. Therefore, it is suggested that psychological characteristics related to falls be summarized and organized for future study.

### 4.2. Additional Items in This Multifactorial Assessment Instrument

Most previous guidelines were developed to describe the past disease history, name of the drugs, and environmental risk of falls in an open ended form question. We tried to organize the list of items correctly to increase the concordance rate of the data analysis even though the person who assesses fall risks varies. This is significant in improving the reliability of this tool compared to other tools.

First, after reflecting on the opinions of experts in various academic fields, new items were added under the disease history factor that had not appeared in previous guidelines. The Delphi panelists thoroughly reviewed the specific factors and items and gave specific opinions on each. Disease history includes these items: stroke, dementia, Parkinson’s, cardiovascular disease, respiratory disease, peripheral neuropathy, diabetes, chronic pain, arthritis, and osteoporosis. Therefore, our study differs from a guideline that includes only a few medical history items such as osteoporosis, depression, and cardiac disease [25]. We identified diseases that affect falls based on evidence and expert opinions and added them to our multifactorial assessment tool.

Regarding the relationship between falls and disease, neurological diseases such as strokes, dementia, Parkinson’s, and peripheral neuropathy are traditionally associated with aging. These conditions might share common cognitive dysfunctions that affect the control of gait and balance [37]. They can limit complex and goal-oriented activities requiring the constant awareness of body movements [38]. Second, some studies identified that cardiovascular diseases in the elderly also increased the risk of falls [39] because the elderly are generally frail with noticeable cognitive decline and multi-morbidity [39]. Similarly, diabetes, arthritis, osteoporosis, and chronic pain are diseases or symptoms with high correlations with the types of fractures that are the most common outcomes of falls [40,41,42].

Moreover, hypoglycemia is the most significant cause of fall episodes [42]. A recent study reported that the adjusted odds of fall-related fractures among patients with hypoglycemic events were 70% higher than in patients without it [43]. These studies consider one explanation to be certain diabetes medications that may increase the risk of fracture and thereby worsen fall-related outcomes [44].

Additionally, arthritis and osteoporosis can lower vitamin D and bone mineral density. Both have been frequently suggested as factors that heighten the risk of bone fracture and falling [40]. Additionally, recent literature reported that elders with multisite pain had a 51% higher chance of fall risk [41]. Research has suggested that those with pain have excessive psychological concerns regarding low balance confidence, reduced self-efficacy of falling, and have mobility limitations such as slower gait pattern and difficulties in activities of daily living (ADL) [45].

Second, we specifically evaluated the use of a wider range of drugs than those included in the existing guidelines—particularly, psychoactive and cardiovascular drugs. Our study included a separate process of sorting and merging related medicines based on the Delphi expert panels. As a result, health care providers received a more comprehensive review of the drugs that affect falls in the elderly. We added those medication names to the medication history factor.

Finally, in our study, experts who participated in Delphi also considered the assessment items related to the residential environment. Based on their recommendations, we added concrete environmental items such as light, carpet, and height of the bed to the residential environment factor.

### 4.3. Limitations and Strengths

Publication limitations may have been present due to the inclusion of English and Korean-only published guidelines. Additionally, our study has a limitation related to validity. Among the methods to confirm the validity of the tool, only expert validity was used. Face validity was not applied. To overcome this problem, we collected the opinions of various fields related to falls and verified validity in various ways by calculating DoCv, and DoCs as well as CV and CVR. This is demonstrated clearly in various factors affecting the falls of the elderly based on worldwide guidelines. Most of the fall risk screening instruments found in the literature tend to focus on one single risk factor [6,46].

Additionally, evidence-based guidelines are developed to assist the practitioner, community residents, and policymaker to make informed clinical decisions [19,47]. Guidelines are valuable resources that play an integral role in improving the intervention and management of various health conditions. We clarified why we extracted each fall risk item based on evidence and expert opinions.

This research gathered all existing factors and filled in missing factors related to falls by collecting various expert opinions. This study increased its validity by adding expert opinions gathered through Delphi studies, in addition to a systematic review method. In this study, the strength of our research was the breadth of expertise within our multidisciplinary panel. These experts thoroughly reviewed the selected guidelines and provided professional opinions on all specific factors and items. Our multifactorial fall risk assessment tool will help to determine proper fall prevention interventions for the elderly in communities.

We clarified why we extracted each fall risk item based on evidence and expert opinions. Conversely, most tools did not describe the criteria for classifying the fall risk items as factors [46,48]. Therefore, the items affecting fall risk that were included in other guidelines were different for each tool. This tool was developed by a thorough, evidence-based approach through the Delphi study and built upon existing guidelines, and so it can be used universally in any country.

All the included guidelines can be internationally used because they did not reflect the situation of a specific country. Therefore, it is necessary to confirm the generalizability of using the tool by identifying whether each multifactorial fall assessment tool has been translated into the language of each country and verifying its validity.

## 5. Conclusions

Health care providers can use comprehensive falls risk screening tools to identify the elderly who are at risk of falling. We developed a multifactorial fall risk assessment tool based on evidence, assessing general characteristics, behavior factors, disease history, medication history, physical function, and environmental factors that reflect the characteristics of the elderly in a community. Although there were existing guidelines, the multifactorial risk factors for falls suggested by each guideline were inconsistent. Therefore, this study attempted to reach a consensus. This study increased the validity of our tool by adding expert opinions gathered through Delphi studies in addition to a systematic review method. This multifactorial fall risk assessment tool, created through this systematic methodology, is expected to be beneficial to the elderly in the community when designing comes to tailored interventions to prevent falls.

## Figures and Tables

**Table 1:** The characteristics of the included studies.

No.	First Author or Publisher (Year)	Country	Age (Years)	Sex	Person Who Performed the Assessment	Factors	Items
1	CDC (2015)	No restrictions	Aged 65 years and over	No restrictions	Health care providers	Biological risk factors	Muscle weakness or balance problems Medication side effects and/or interactions Chronic health conditions such as arthritis and stroke Vision changes and vision loss Loss of sensation in feet
						Behavioral risk factors	Inactivity Risky behaviors such as standing on a chair in place of a step stool Alcohol use
						Environmental risk factors	Clutter and tripping hazards Poor lighting Lack of stair railings Lack of grab bars inside and outside the tub or shower Poorly designed public spaces
2	Avin. K.G. (2015)	No restrictions	Aged 65 years and over	No restrictions	Physical therapist	Medication review with emphasis on polypharmacy and psychoactive drugs	
						Medical history with an emphasis on new or unmanaged risk factors	Osteoporosis Depression Cardiac disease, including signs or symptoms of cardioinhibitory carotid sinus hypersensitivity
						Body functions and structure, activity and participation, environmental factors, and personal factors	Strength Balance Gait Activities of daily living Footwear Environmental hazards Cognition Neurological function Cardiac function, including postural hypotension Vision Urinary incontinence
3	Canada PHAC (2014)	Canada	Aged 65 years and over	No restrictions	N/I	Biological or intrinsic risk factors	Acute illness Balance and gait deficits Chronic conditions and disabilities Cognitive impairments Low vision Muscle weakness and reduced physical fitness
						Behavioral risk factors	Assistive devices Excessive alcohol Fear of falling Footwear and clothing History of previous falls Inadequate diet Medications Risk-taking behavior Vitamin D
						Social and economic risk factors	Social networks Socio–economic status:
						Environmental risk factors	Factors in the community Factors in the living environment Weather and climate
4	ACSQHC (2009)	Australia	Aged 65 years and over	No restrictions	Health professionals, and all members of the health care team	Intrinsic risk factors	Increased age History of falls Chronic medical conditions (e.g., stroke, Parkinson’s disease, arthritis) Multiple medications and specific types (e.g., psychoactive drugs) Impaired balance and mobility Reduced muscle strength Sensory problems (e.g., impaired vision, peripheral neuropathy) Dizziness Impaired cognition Incontinence Depression Low levels of physical activity Slow reaction time Fear of falling Being female
						Extrinsic risk factors	Inappropriate footwear (high heels and slippers) Inappropriate spectacles Hazards inside and outside the home
5	BC, Ministry of Health (2004)	British Columbia	Aged 65 years and over	No restrictions	Community health workers, home care nurses, and other senior service providers	Biological/medical risk factors	Advanced age Gender Chronic and acute illness Physical disability Muscle weakness and diminished physical fitness Vision changes Cognitive impairments
						Behavioral risk factors	Risk-taking behaviors Medication use Inattention Alcohol use Inappropriate footwear Handbags Inadequate diet/exercise Fear of falling
						Environmental risk factors	Home hazards Community hazards Institutional hazards
						Social and economic risk factors	
6	WHO (2004)	No restrictions	Aged 65 years and over	No restrictions	Emergency department medical staff, health authorities, primary health care teams,	Intrinsic risk factors	A history of falls, age, gender (women), living alone, ethnicity, medicines, medical conditions (circulatory disease, chronic obstructive pulmonary disease, depression, and arthritis, chronic disease burden, thyroid dysfunction, dizziness, depression, and incontinence), impaired mobility and gait, sedentary behavior, psychological status, nutritional deficiencies, impaired cognition, visual impairments, foot problems
						Extrinsic risk factors	Environmental hazards (poor lighting, slippery floors, uneven surfaces, etc.) Footwear and clothing Inappropriate walking aids or assistive devices
7	Washington State Department of Health (2002)	No restrictions	Aged 65 years and over	No restrictions	A nurse or other health professional trained to conduct tests	Demographic characteristics of people who fall	Age (65 years or older) Gender (female) Race (White)
						Causes of falls	Chronic health problems Physical and functional impairments Alcohol and medication use Hazards in the home
8	HSE (2008)	Ireland	Aged 65 years and over	No restrictions	N/I	Intrinsic risk factors	Muscle weakness History of falls Gait and balance deficits Visual deficits Arthritis Depression Cognitive impairment Age > 80 years Urinary incontinence Orthostatic or postprandial hypotension Dizziness Fear of falling Limited activity (institutional setting) Hearing (institutional setting)
						Extrinsic risk factors	Use of assistive devices Impaired ADL High level of activity (community setting) Medication
						Environmental risk factors	Environmental hazards Home hazards
9	USPSTF (2018)	United States of America	Aged 65 years and over	No restrictions	Clinicians (usually nursing staff)	Biological factors	Age Physical function Mobility limitation
						Behavioral factor	A history of falls

Notes: CDC = Centers for Disease Control and Prevention; PHAC = Public Health Agency of Canada; ACSQHC = Australian Commission on Safety and Quality in Health Care; BC = British Columbia; WHO = World Health Organization; HSE = Health Service Executive; USPSTF = United States Preventive Services Task Force; ADL = activities of daily living.

**Table 2:** Results of the Appraisal of Guidelines for Research and Evaluation II (AGREE II) evaluation.

	Guideline Development Group									
Domain	CDC	Avin et al.	Canada PHAC	ACSQHC	BC, Ministry of Health	WHO	Washington State Department of Health	HSE	USPSTF	Mean (Range), %
1. Scope and Purpose	83.3	100	83.3	100.0	83.3	44.4	83.3	83.3	85.7	83.0 (44.4–100.0)
2. Stakeholder Involvement	77.8	77.8	44.4	66.7	50.0	55.6	55.6	44.4	85.7	62.0 (44.4–85.7)
3. Rigor of Development	29.2	81.3	27.1	85.4	25.0	37.5	25.0	45.8	82.1	48.7 (25.0–85.4)
4. Clarity of Presentation	88.9	50.0	44.4	88.9	61.1	33.3	55.6	44.4	81.0	60.8 (33.3–88.9)
5. Applicability	50.0	0	50.0	50.0	25.0	25.0	75.0	25.0	28.6	36.5 (0–75.0)
6. Editorial Independence	33.3	83.3	0	83.3	33.3	33.3	50.0	0	78.6	43.9 (0–83.3)
Overall Outcome of Guideline Development	66.7	66.7	66.7	100.0	66.7	66.7	66.7	66.7	73.8	71.2 (66.7–100.0)

Notes: CDC = Centers for Disease Control and Prevention; PHAC = Public Health Agency of Canada; ACSQHC = Australian Commission on Safety and Quality in Health Care; BC = British Columbia; WHO = World Health Organization; HSE = Health Service Executive; USPSTF = United States Preventive Services Task Force.

**Table 3:** Summary of factors and items suggested by the systematic reviews.

Factors	Behavioral Factor	Biological Factor	Environmental Factor	General Factor
Items	Multiple medication use Excess alcohol intake Lack of exercise Inadequate diet History of previous falls Fear of falling Inappropriate footwear Use of assistive devices	Sex (Female) Increased age Impaired ADL Low vision History of disease Musculoskeletal function Mobility/balance/gait deficits Neurological function Cognitive capacity Cardiac function Cardiovascular drugs Psychoactive drugs Vitamin D deficiency Incontinence Hypotension Dizziness Medication side effect	Indoor environment Outdoor environment Social network	Low income Living alone

Notes: ADL: activities of daily living.

**Table 4:** Results of the scoring round Delphi survey and the final items of the MFA-C.

Factors	Items	2nd Round							3rd Round								Judgment	
		Necessity				Applicability			Necessity				Applicability					
		CVR	DoCs	DoCv	CV	CVR	DoCs	DoCv	CV	CVR	DoCs	DoCv	CV	CVR	DoCs	DoCv	CV	
General Characteristics	Sex (female)	1.00	1.00	0.00	0.00	1.00	1.00	0.00	0.00	1.00	1.00	0.00	0.00	1.00	1.00	0.00	0.00	Included
	Increased age	1.00	1.00	0.00	0.00	1.00	1.00	0.00	0.00	1.00	1.00	0.00	0.00	1.00	1.00	0.00	0.00	Included
	Living alone	1.00	1.00	0.00	0.07	1.00	1.00	0.00	0.00	1.00	1.00	0.00	0.07	1.00	1.00	0.00	0.00	Included
	Low income	1.00	0.80	0.50	0.16	0.80	0.50 *	0.50	0.16	1.00	0.80	0.50	0.11	1.00	0.80	0.50	0.11	Included (after discussion)
Behavior Factor	Inadequate diet	1.00	0.80	0.50	0.20	1.00	0.80	0.50	0.16	1.00	0.75	0.50	0.11	1.00	0.80	0.50	0.10	Included
	History of previous falls	1.00	1.00	0.00	0.00	1.00	1.00	0.00	0.07	1.00	1.00	0.00	0.00	1.00	1.00	0.00	0.06	Included
	Fear of falling	1.00	1.00	0.00	0.07	1.00	1.00	0.00	0.09	1.00	1.00	0.00	0.00	1.00	1.00	0.00	0.07	Included
	Lack of exercise	1.00	1.00	0.00	0.09	1.00	0.80	0.50	0.12	1.00	1.00	0.00	0.00	1.00	0.80	0.50	0.11	Included
	Vitamin D deficiency	1.00	0.80	0.50	0.20	1.00	0.60 *	1.00 *	0.23									Excluded
	Excess alcohol intake	1.00	1.00	0.00	0.07	1.00	1.00	0.00	0.17	1.00	1.00	0.00	0.07	1.00	1.00	0.00	0.09	Included
Disease History	Stroke	1.00	1.00	0.00	0.00	1.00	1.00	0.00	0.00	1.00	1.00	0.00	0.00	1.00	1.00	0.00	0.00	Included
	Dementia	1.00	1.00	0.00	0.00	1.00	1.00	0.00	0.00	1.00	1.00	0.00	0.00	1.00	1.00	0.00	0.00	Included
	Parkinson’s	1.00	1.00	0.00	0.00	1.00	1.00	0.00	0.00	1.00	1.00	0.00	0.00	1.00	1.00	0.00	0.00	Included
	Dizziness	1.00	1.00	0.00	0.00	1.00	1.00	0.00	0.00	1.00	1.00	0.00	0.00	1.00	1.00	0.00	0.00	Included
	Cardiovascular	1.00	1.00	0.00	0.00	1.00	1.00	0.00	0.00	1.00	1.00	0.00	0.07	1.00	0.80	0.50	0.19	Included
	Hypotension	1.00	1.00	0.00	0.00	1.00	1.00	0.00	0.07	1.00	1.00	0.00	0.00	1.00	1.00	0.00	0.00	Included
	Respiratory	1.00	1.00	0.00	0.00	1.00	1.00	0.00	0.00	1.00	1.00	0.00	0.00	1.00	1.00	0.00	0.00	Included
	Peripheral neuropathy	1.00	1.00	0.00	0.00	1.00	1.00	0.00	0.15	1.00	1.00	0.00	0.00	1.00	1.00	0.00	0.09	Included
	Diabetes	1.00	1.00	0.00	0.00	1.00	1.00	0.00	0.00	1.00	0.95	0.13	0.16	1.00	0.95	0.13	0.15	Included
	Chronic pain	1.00	1.00	0.00	0.00	1.00	1.00	0.00	0.00	1.00	1.00	0.00	0.00	1.00	1.00	0.00	0.06	Included
	Arthritis	1.00	1.00	0.00	0.00	1.00	1.00	0.00	0.00	1.00	1.00	0.00	0.00	1.00	1.00	0.00	0.00	Included
	Osteoporosis	1.00	1.00	0.00	0.00	1.00	1.00	0.00	0.07	1.00	1.00	0.00	0.00	1.00	1.00	0.00	0.00	Included
	Incontinence	1.00	0.80	0.50	0.17	1.00	0.75	0.63 *	0.21	1.00	0.80	0.50	0.10	1.00	0.80	0.50	0.11	Included (After discussion)
Medication History	Psychoactive drugs	0.93	1.00	0.00	0.08	0.93	1.00	0.00	0.12	1.00	1.00	0.00	0.04	1.00	1.00	0.00	0.04	Included
	Cardiovascular drugs	1.00	0.98	0.06	0.08	1.00	1.00	0.00	0.08	1.00	1.00	0.00	0.00	1.00	1.00	0.00	0.00	Included
	Multiple medication use	1.00	0.80	0.50	0.17	1.00	0.80	0.50	0.11	1.00	0.95	0.13	0.10	1.00	0.95	0.13	0.09	Included
	Medication side effects	0.88	0.88	0.25	0.26	0.63 *	0.25 *	1.13 *	0.47									Excluded
Physical Function	Low vision	1.00	1.00	0.00	0.08	1.00	1.00	0.00	0.00	1.00	1.00	0.00	0.00	1.00	1.00	0.00	0.00	Included
	Mobility/balance/gait deficits	1.00	0.95	0.12	0.10	1.00	0.95	0.13	0.10	1.00	1.00	0.00	0.08	1.00	1.00	0.00	0.07	Included
	Impaired ADL	1.00	0.90	0.25	0.17	1.00	0.95	0.25	0.17	1.00	1.00	0.00	0.07	1.00	0.90	0.25	0.10	Included
	Musculoskeletal function	1.00	1.00	0.00	0.07	1.00	0.95	0.13	0.10	1.00	1.00	0.00	0.07	1.00	0.95	0.13	0.09	Included
	Cardiac function	1.00	0.98	0.06	0.12	1.00	0.58 *	1.00 *	0.24	1.00	1.00	0.00	0.07	1.00	0.80	0.50	0.19	Included (After discussion)
	Neurological function	1.00	1.00	0.00	0.00	1.00	0.92	0.20	0.18	1.00	1.00	0.00	0.00	1.00	1.00	0.00	0.02	Included
	Inappropriate footwear	1.00	0.95	0.13	0.10	1.00	0.95	0.13	0.10	1.00	1.00	0.00	0.07	1.00	1.00	0.00	0.09	Included
	Use of assistive devices	1.00	1.00	0.00	0.00	1.00	1.00	0.00	0.00	1.00	1.00	0.00	0.07	1.00	1.00	0.00	0.00	Included
Cognitive Function	Cognitive capacity	0.93	0.88	0.31	0.17	0.93	0.60 *	1.00 *	0.15									Excluded
Environmental Factor	Indoor environment	1.00	1.00	0.00	0.08	1.00	1.00	0.00	0.08	1.00	0.97	0.07	0.07	1.00	1.00	0.01	0.03	Included
	Outdoor environment	1.00	1.00	0.00	0.11	1.00	1.00	0.00	0.22	1.00	1.00	0.00	0.07	1.00	0.98	0.04	0.08	Included
	Social network	1.00	1.00	0.00	0.15	1.00	1.00	0.00	0.14	1.00	1.00	0.00	0.15	1.00	1.00	0.00	0.15	Included

Notes: MFA-C = Multifactorial Fall Risk Assessment Tool for Community-Dwelling Older People; CVR = content validity ratio; DoCs = degree of consensus; DoCv = degree of. convergence; CV = coefficient of variation; ADL = activities of daily living. * exclusion criteria: CVR< 0.78, DoCs < 0.75, DoCv > 0.50, CV ≥ 0.8.

**Table 5:** MFA-C.

Factors	Items	Contents of Question	Options			
General characteristics	Sex (female)	Sex (female)	Male	Female		
	Increased age	Age	Age			
	Living alone	Residential type	Alone	Together		
	Low income	Health insurance	Medical insurance	Medicaid 1	Medicaid 2	
Behavior factor	Inadequate diet	Number of meals/day	3 times of meal/day	2 times of meal/day	1 time of meal/day	Poor, irregular
	History of previous falls	Experience of falls	Yes (experienced)	No (inexperienced)		
		Details of fall experience	Time	Place	Number of falls	Extent of damage
	Fear of falling	Going out alone	Feeling no fear	Feeling like usual	Feeling a little fear	Feeling a lot of fear
		Cooking alone	Feeling no fear	Feeling like usual	Feeling a little fear	Feeling a lot of fear
		Activities in the bathroom	Feeling no fear	Feeling like usual	Feeling a little fear	Feeling a lot of fear
		Getting out of bed alone	Feeling no fear	Feeling like usual	Feeling a little fear	Feeling a lot of fear
		Walking for exercise	Feeling no fear	Feeling like usual	Feeling a little fear	Feeling a lot of fear
		Going out on a slippery road (snow, rain, frozen road)	Feeling no fear	Feeling like usual	Feeling a little fear	Feeling a lot of fear
		Visiting friends or relatives alone	Feeling no fear	Feeling like usual	Feeling a little fear	Feeling a lot of fear
		Lowering things on the head	Feeling no fear	Feeling like usual	Feeling a little fear	Feeling a lot of fear
		Going to crowded places	Feeling no fear	Feeling like usual	Feeling a little fear	Feeling a lot of fear
		Going up and down the stairs	Feeling no fear	Feeling like usual	Feeling a little fear	Feeling a lot of fear
		Bending over and grabbing objects	Feeling no fear	Feeling like usual	Feeling a little fear	Feeling a lot of fear
	Lack of exercise	Times of exercise/day	None	< 30 min	30 min–1 h	1–2 h
			> 2 h			
	Excess alcohol intake	Alcohol intake	Yes	No	Stop drinking	
		Details of alcohol intake	Kind of alcoholic drink	Average drinking quantity	A period of drinking	
Disease history	Stroke	Having a disease	Yes	No		
	Dementia	Having a disease	Yes	No		
	Parkinson’s	Having a disease	Yes	No		
	Dizziness	Having a disease	Yes	No		
	Cardiovascular	Having a disease	Yes	No		
	Hypotension	Having a disease	Yes	No		
	Respiratory	Having a disease	Yes	No		
	Peripheral neuropathy	Having a disease	Yes	No		
	Diabetes	Having a disease	Yes	No		
	Chronic pain	Having a disease	Yes	No		
	Arthritis	Having a disease	Yes	No		
	Osteoporosis	Having a disease	Yes	No		
	Incontinence	Having a disease	Yes	No		
Medication history	Psychoactive drugs	Taking sedative drugs	Yes	No		
		- Diazepam	Yes	No		
		- Etizolam	Yes	No		
		- Clonazepam	Yes	No		
		- Lorazepam	Yes	No		
		- Alprazolam	Yes	No		
		Taking haloperidol	Yes	No		
		Taking sleeping drugs				
		- Zolpidem	Yes	No		
		Taking antiemetic drugs	Yes	No		
		Taking antidepressants				
		- TCAs	Yes	No		
		- SSRIs	Yes	No		
	Cardiovascular drugs	Taking loop diuretics	Yes	No		
		Taking antiarrhythmic drugs	Yes	No		
		Taking digoxin	Yes	No		
		Taking oral hypoglycemic/insulin	Yes	No		
		Taking calcium channel blockers	Yes	No		
	Multiple medication use	Total number of medication	≤ 3	4	5	≥ 6
Physical function	Low vision	Eyesight	Left eyesight	Right eyesight	Unknown	
		Wearing glasses	Yes	No		
		Diabetic retinopathy	Yes	No		
		Ophthalmologic disease	Yes	No		
	Mobility/balance/gait deficits	30 s chair stand test below-average score based on age and gender	Age; 60–64	Men: <14	Women: <12	
		- Average score	Age; 65–69	Men: <12	Women: <11	
			Age; 70–74	Men: <12	Women: <10	
			Age; 75–79	Men: <11	Women: <10	
			Age; 80–84	Men: <10	Women: <9	
		4-step balance test within 10 s	Yes	No		
		- Standing upright				
		- Standing aside				
		- Tandem gait				
		- Standing on one leg				
		Taking TUG test more than 12 s	Yes	No		
	Impaired ADL	Bathing	Dependence	Partial dependence	Independence	
		Dressing	Dependence	Partial dependence	Independence	
		Using the toilet	Dependence	Partial dependence	Independence	
		Transferring	Dependence	Partial dependence	Independence	
		Continence	Dependence	Partial dependence	Independence	
		Feeding	Dependence	Partial dependence	Independence	
	Musculoskeletal function	Restriction of ROM				
		- Upper limbs	Yes	No		
		- Lower limbs	Yes	No		
		- Hip joint	Yes	No		
		- Knee joint	Yes	No		
		- Ankle joint	Yes	No		
	Cardiac function	Heart rate	Heart rate (/min)			
		Arrhythmia	Yes	No		
		- Result of EKG				
		Postural hypotension	Yes	No		
			Standing position (BP/HR)	Supine position (BP/HR)	Standing position (BP/HR)	
	Neurological function	Disease history				
		- CVA	Yes	No		
		- Epilepsy or seizure	Yes	No		
		- Walk-related diseases	Yes	No		
		- Peripheral neuropathy	Yes	No		
		- Peripheral vertigo	Yes	No		
	Inappropriate footwear	Toe deformities/ulcer	Yes	No		
	Use of assistive devices	Walking assistance device	Yes	No		
		Power train (e.g., wheelchair)	Yes	No		
Environmental factor	Indoor environment	Risk factors in the living room and bedroom				
		- Brightness of light	Brightness	Normal	Darkness	Lux
		- Bare and telephone wire	Yes	No		
		- Carpet	Yes	No		
		- Slipperiness	Yes	No		
		- Height of threshold	High	Medium	Low	None
		- Height of bed	High	Medium	Low	None
		Risk factors of bathroom				
		- Brightness of light	Brightness	Normal	Darkness	Lux
		- Slipperiness	Yes	No		
		- Nonslip mat	Yes	No		
		- Height of threshold	High	Medium	Low	None
		- Safety rail of shower booth	Yes	No		
	Outdoor environment	Risk factors of outdoor environment				
		- Brightness of light	Brightness	Normal	Darkness	Lux
		- Access road	Slipperiness	The steep slope of a footpath	Broken sidewalk block	No elevator
		- Height of stairs	High	Medium	Low	Damaged stairs
			None			
		- Safety rail	Yes	No		
	Social network	Support of community	Yes	No		

Notes: ADL = activities of daily living; TCAs = tricyclic antidepressants, SSRIs = selective serotonin reuptake inhibitors; TUG = time up and go test; ROM = range of motion; EKG = electrocardiogram, BP = blood pressure; HR = heart rates; CVA = cerebrovascular accident.

## Supplementary Materials

The following are available online at https://www.mdpi.com/1660-4601/17/17/6097/s1, Table S1: Search term, Figure S1: Previous flow chart.
