# Supplementary material for: A Systematic Review of the Guidelines and Delphi Study for the Multifactorial Fall Risk Assessment of Community-Dwelling Elderly
Source: Int J Environ Res Public Health. 2020 Aug 21;17(17):6097. doi: 10.3390/ijerph17176097 (PMC7504076; doi:10.3390/ijerph17176097)
Supplement: Supplementary file 1 [file ijerph-17-06097-s001.pdf]

**Supplementary S1**

**Table S1.** Search term

| | |
|---|---|
| | **Ovid-MEDLINE, Cochrane Library** |
| **1** | (senior* or elder* or old* or older or ag*ing or old-age*).tw. |
| **2** | exp Aged/ |
| **3** | or/1-2 |
| **4** | (fall or Falls or faller* or falling or fell or slip$ or stumble$ or tumble$).mp. |
| **5** | Accidental Falls/ |
| **6** | or/4-5 |
| **7** | assessment*.mp. |
| **8** | Geriatric Assessment/ |
| **9** | Needs Assessment/ |
| **10** | exp Risk Assessment/ |
| **11** | Self-Assessment/ |
| **12** | or/7-11 |
| **13** | evaluation*.mp. |
| **14** | Evaluation Studies/ |
| **15** | or/13-14 |
| **16** | guide*.mp. |
| **17** | exp Guideline |
| **18** | or/16-17 |
| **19** | (multifactorial or multifacted).mp. |
| **20** | 6 and (12 or 15 or 18) and 19 |
| **21** | and/3, 20 |
| | **EMBASE** |
| 1 | (senior* or elder* or old* or ag*ing or old-age*).mp. |
| 2 | exp Aged/ |
| 3 | or/1-2 |
| 4 | (fall* or fell or slip* or stumble* or tumble*).mp. |
| 5 | Falling/ |
| 6 | Fall Risk/ |
| 7 | Home Accident/ |
| 8 | or/4-7 |
| 9 | assessment*.mp. |
| 10 | Fall Risk Assessment/ |
| 11 | Community Assessment/ |
| 12 | Risk Assessment/ |
| 13 | Geriatric Assessment/ |
| 14 | or/9-13 |
| 15 | evaluation*.mp. |
| 16 | Evaluation Study/ |
| 17 | Self-Evaluation/ |
| 18 | or/15-17 |
| 19 | guide* |
| 20 | exp Practice Guideline/ |
| 21 | or/19-20 |
| 22 | (multifactorial or multifaceted).mp. |
| 23 | 8 and (14 or 18 or 21) and 22 |
| 24 | and/3, 23 |
| | **Trip database** |

| | |
|---|---|
| 1 | elderly or older or aged or elder or old-age |
| 2 | falls and (assessment or evaluation or guideline or multifactorial) |
| 3 | and/1-2 |
| **Guideline International Network, National Guideline Clearing House, WHO, CDC** | |
| 1 | falls |

Notes: WHO= World Health Organization. HSE= Health Service Executive. CDC= Centers for Disease Control and Prevention. RISS= Research Information Sharing Service. KISS= Korean Studies Information Service. KM base= Korean Medical Database

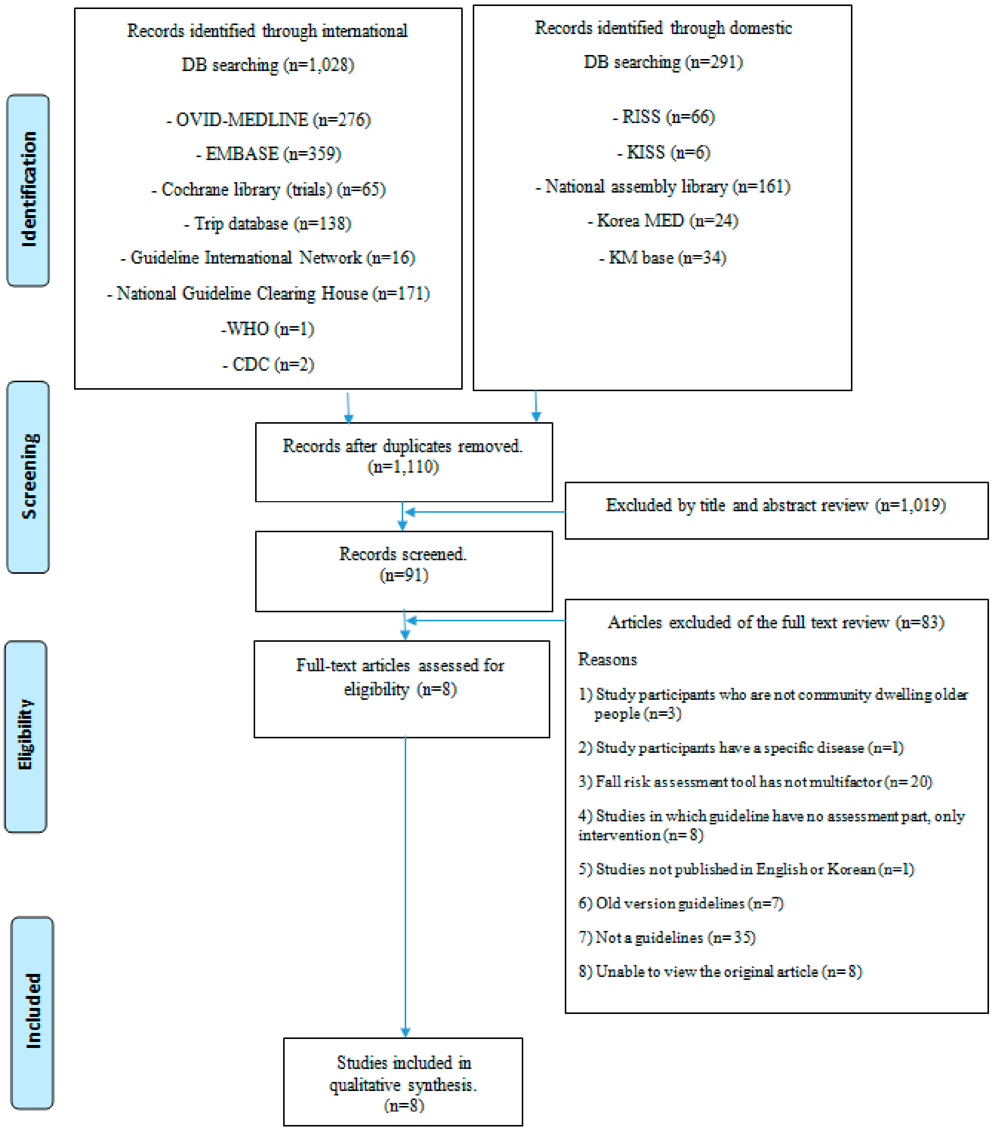


**Figure S1.** Previous flow chart
